# Assessing the need for a doctor of philosophy (Ph.D.) degree in Endodontics: perspective and implication for advancing dental education and research in Saudi Arabia

**DOI:** 10.1186/s12909-024-06485-w

**Published:** 2024-12-18

**Authors:** Loai Alsofi

**Affiliations:** Department of Endodontics, Faculty of Dentistry, King Abdulaziz University, P.O. Box 80209, Jeddah, 21589 Saudi Arabia

**Keywords:** Continuing Dental Education, Dental Research, Endodontic Research, Endodontic Teaching, Ph.D. Program, Professional Saudi Arabia

## Abstract

**Aim:**

This study aims to evaluate the necessity and feasibility of integrating a Doctor of Philosophy (Ph.D.) degree into Endodontic programs at the Faculty of Dentistry King Abdulaziz University, (KAUFD), Jeddah, Saudi Arabia, and to gather insights from key stakeholders regarding the value and necessity of a Ph.D. in enhancing the field of Endodontics.

**Materials and Methods:**

An online questionnaire link was distributed among potential Ph.D. candidates and key decision-makers in Endodontics in Saudi Arabia through social media and emails to members of the Saudi Endodontic Society and the Saudi Endodontic Journal. The questionnaire assessed demographics, professional background, interest in Ph.D. programs, program preferences, perceived need for Ph.D. programs in various sectors, and university selection criteria. Data were analyzed using IBM SPSS Statistics (Version 23).

**Results:**

141 responses were received. Most respondents were aged 31–40, with an almost equal gender distribution. In terms of current position, a significant number were Endodontic consultants (35.7%) and Endodontic residents/Master students (28.6%) (*P* = 0.006). Additionally, a considerable proportion of probable prospective students had less than 5 years of expertise (45.7%) (*p* = 0.013), and the highest degree held by many was a bachelor’s degree (35.7%) (*P* = 0.007). Key considerations for prospective students were program duration, Saudi Commission for Health Specialists (SCFHS) recognition, part-time options, and cost. The importance of acquiring research skills, such as learning new research technologies and gaining publication experience, was also highlighted.

**Conclusion:**

The findings strongly suggest a need for a Ph.D. program in Endodontics in Saudi Arabia. The results provide valuable insights for developing a program that aligns with potential candidates’ and key stakeholders’ needs and preferences, thereby advancing Endodontic research and education within the country.

**Supplementary Information:**

The online version contains supplementary material available at 10.1186/s12909-024-06485-w.

## Introduction

Endodontics is a branch of dentistry that deals with the etiology, diagnosis, prevention, and treatment of diseases of the pulp and periapical tissues compatible with good health [1]. Endodontics as a specialty has undergone significant advancements in recent years [2]. This considerable advancement includes the introduction of magnification devices, engine-driven file systems, materials for regeneration, and Cone Beam Computed Tomography (CBCT). This progress contributes to a shift in the understanding and satisfaction of endodontic specialties [3].

General practitioners are the first line of dental care who provide endodontic treatment for patients who are in need [4, 5]. However, cross-sectional studies show that the quality of root canal treatment performed by general practitioners is substandard or inadequate [6–10]. This can be caused by either the complexity of the cases or the graduation of inexperienced practitioners [11, 12]. The introduction of modern technologies in the field, such as nickel-titanium rotary files, bioceramic sealers, and magnification devices made root canal treatment procedures simpler and more predictable [11, 13–16].

As the field develops, the need for highly qualified professionals who can contribute to clinical practice and academic research becomes crucial. The current dental education system in Saudi Arabia primarily focuses on undergraduate Bachelor’s degrees, Master’s degree programs, and specialized training residency programs (Saudi Board in Endodontics) [17], which provide a solid foundation in clinical practices.

In the meantime, there is a gap in integrating advanced research training and academic development at the doctoral level within the endodontic specialty. This has led to many debates about the possible benefits of introducing a Doctor of Philosophy (Ph.D) degree specifically designed for Endodontics. In Saudi Arabia, the landscape of dental education is rapidly exchangeable, reflecting global trends and the increased complexity of dental care. This necessitates reevaluating the educational pathways available to aspiring endodontists, particularly the potential introduction of a Ph.D. degree within Endodontics programs. The demand for doctoral programs is evident in other health-related specialties. A study by Badreldin et al. in 2022 showed that 80% of respondents felt the establishment of a Doctor of Pharmacy/Master of Public Health Dual Degree in Saudi Arabia would increase their job opportunities [18].

The integration of a Ph.D. program in Saudia Arabia could address several pressing needs within the field. First, it would foster a culture of research and innovation, essential for the advancement of endodontic practices. As new techniques, materials, and technologies emerge, having a cadre of well-trained researchers is crucial to evaluate and implement these advancements effectively [3]. A Ph.D. program would equip graduates with the skills necessary to conduct stringent scientific research, critically analyze existing literature, and contribute original findings that could enhance patient outcomes, and elevate the standards of care in Endodontics. Moreover. a Ph.D. program would emphasize the importance of research literacy, enabling future endodontists in Saudi Arabia to become not only skilled clinicians but also reasoning leaders in the field. This is particularly important in a country where the dental health needs are progressively developing and there is a growing emphasis on preventive care and the management of complex cases. Furthermore, the international dental landscape is progressively affected by evidence-based practice, requiring practitioners to stay side by side with current research and incorporate it into their clinical decision-making.

Shetty et al., 2021, did a university-based survey of career choices in higher education among dental students in the United Arab Emirates (UAE) [19]. They concluded that dental undergraduate students in the UAE prefer to pursue specialization in clinical branches like Orthodontics and Endodontics. Moreover, the survey respondents felt that there was a need to offer more dental post-graduate and dental ph.D. programs by UAE universities. Another study was conducted in the College of Dentistry, Ajman University to assess the perception and understanding of the Endodontic specialty among dental students and newly graduated dentists. It also evaluated their interest in pursuing Endodontics as a future career. Results indicated that Ajman University dental students and interns showed a satisfactory perception of the Endodontic specialty. Also, perception did not significantly differ based on gender, level of study, or the experience of the supervising instructor [20]. Contrary to these results, Newton et al. 2000 assessed whether differences existed between male and female dental clinicians in the types of positions they occupy in the United Kingdom [21]. Their findings indicated that most females are employed in general dental practice. Another study made by Chan et al. 2006 concluded that 95.8% of the 386 general dentists who participated in the 26th Asia Pacific Dental Congress declared that they would take continuing professional dental education programs and courses within the next 5 years [22].

In 2016, Puryer, Kostova, and Kouznetsova conducted a cross-sectional survey to explore the attitudes toward postgraduate specialization of final-year students at one UK dental school and to identify possible influencing factors. The results indicated that 6.9% of undergraduates had an intention to specialize in Endodontics as their first choice of specialty and 17.2% as their second choice of specialty [23]. Age and ethnicity did not affect the intention to specialize. Gender influences were more apparent among females who did not wish to specialize [23]. Al-Dlaigan et al., 2011 studied the career development of males who graduated from King Saud University (KSU) in terms of the pursuit of postgraduate dental education, higher degree, or Board certification after qualification using a questionnaire survey. They concluded that most of the male dentists who graduated from the College of Dentistry, KSU, were motivated and eager to continue their postgraduate education to get higher academic degrees from nationally or internationally well-recognized universities to improve their career and self-esteem [24]. In another study, Al-Dlaigan et al., 2012 investigated the number of female bachelor of dental surgery graduates who earned a postgraduate education degree from KSU and a degree of qualification using a questionnaire survey. They concluded that 54% of the female dentists had pursued postgraduate education. Out of these 5% had Saudi board, 5% had obtained degrees in Endodontics, 59% had a master’s degree, 7% had a doctorate, and 2% had fellowship certificates [25]. lrashdan et al.2018, explored the professional career plans among final-year dental students from different backgrounds at a single Middle Eastern institution using a self-administered questionnaire. Results concluded that several differences in career plans were found between dental students from variable backgrounds studying a single institution [26]. Many of these disparities could reflect variations in socioeconomic backgrounds. A cross-sectional study across seven dental colleges in Saudi Arabia by Fita et al. 2020, assessed dental students’ opinions about their future career challenges in the dental profession and factors associated with the perception of career challenges. It was concluded that establishing a private clinic and getting a government job were the most common career challenges [27]. Senior students, female students, and students with low academic scores had an increased likelihood of facing employment and academic-related difficulties [27].

The importance and value of establishing a research-based program in the field of Endodontics need further assessment of its feasibility and desirability by potential candidates and key stakeholders. Such a program will provide dental schools with highly educated faculty members who can provide better clinical training for graduate and undergraduate students and write and develop Endodonitc curricula that meet national and international demands.

This study aims to evaluate the necessity and feasibility of integrating a Doctor of Philosophy (Ph.D) degree into the endodontic programs at King Abdulaziz University Faculty of Dentistry (KAUFD), in Saudi Arabia. In addition, we aim to gather insights from key stakeholders including practitioners and students to understand their perspectives on the value and necessity of a Ph.D. in enhancing the field of Endodontics leading to improved patient care and health outcomes.

## Materials and methods

The research was conducted in accordance with the Declaration of Helsinki. The research was approved by the Ethics Research Committee at the Faculty of Dentistry, King Abdulaziz University (Propocal No: 84-09-24).

We conducted an online survey to evaluate the need for and design considerations of Ph.D. programs in Endodontics in Saudi Arabia. The survey targeted potential Ph.D. candidates and key decision-makers in the field of Endodontics and was distributed through social media and emails to members of the Saudi Endodontic Society and the Saudi Endodontic Journal. We used a structured questionnaire (Appendix A), which was developed based on a literature review and expert consultation.

The questionnaire was designed in the form of closed questions, as a ‘‘tick box’’ that included 14 multiple choice questions. The validity of the questions was evaluated by several Endodontic faculty members to establish the reliability and validity of the questions. The Delphi method was used during the process of developing the questionnaire [28, 29]. The final version of the questionnaire was approved by the Ethics Research Committee at the Faculty of Dentistry, King Abdulaziz University.

The sample size was estimated based on the WHO recommendations. The required sample size should be equal to or greater than 125 participants to achieve a 95% confidence interval and a 5% significance level (p-value), estimated using OpenEpi VER 3.0 [30].

The questionnaire included sections on demographics, professional background, interest in Ph.D. programs, program preferences, perceived need for Ph.D. programs in various sectors (assessed using a 5-point Likert scale), and university selection criteria. We developed the survey using Google Forms and distributed the survey link via email, social media, and professional networks.

### Statistical analysis

The data were analyzed using IBM SPSS Statistics (Version 23; Armonk, NY: IBM Corp.). Descriptive statistics, including frequencies and percentages, were calculated for demographic variables and categorical responses. Continuous variables were reported as means and standard deviations. For inferential statistics, the chi-square test of independence was used to examine the relationships between categorical variables and the Mann-Whitney U tests for Likert scale. A significance level of 0.05 was used for all statistical tests. P-values less than 0.05 were considered indicative of statistically significant differences.

## Results

We received 141 responses for the survey. According to the regulations of the Ministry of Education in Saudi Arabia, one must have a master’s degree in Endodontics from an accredited program. The demographics of the participants, as shown in (Table 1), indicate that the majority of respondents are in the age range of 31–40 years (44.0%), with an almost equal gender distribution (52.5% male, 47.5% female). A significant part holds a Master’s degree (29.1%) or a Bachelor’s degree (24.8%), and the largest group among the current level of dental education had board certification (45.4%). According to the Saudi Commission for Health Specialists (SCFHS) classification, participants were mostly Consultants (44.0%), and the most common place of work was Government hospitals (51.8%).

**Table 1:** Demographic Characteristics of Participants (*N* = 141)

Variables	% (*N*)
**Age**	
- 20–30	27.7% (39)
- 31–40	44.0% (62)
- 41–50	17.7% (25)
- 51–60	10.6% (15)
**Gender**	
- Male	52.5% (74)
- Female	47.5% (67)
**Current level of dental education**	
- Undergraduate student	5.0% (7)
- General dentist	5.7% (8)
- Endodontic resident/Master student	17.0% (24)
- Master’s Degree	23.4% (33)
- Board Certification	45.4% (64)
- Doctorate Degree	3.5% (5)
**What is your current SCFHS classification**	
- General dentist	25.5% (36)
- Registrar	12.8% (18)
- Senior registrar	10.6% (15)
- Consultant	44.0% (62)
- NA	7.1% (10)
**Place of work**	
- Government University	36.2% (51)
- Private University	2.8% (4)
- Private hospital	9.2% (13)
- Government hospital	51.8% (73)
**Years of expertise**	
- < 5 years	35.5% (50)
- < 10 years	16.3% (23)
- 10–15 years	27.7% (39)
- 16–20 years	8.5% (12)
- > 20 years	12.1% (17)
**Highest degree**:	
- Bachelor’s degree	24.8% (35)
- Masters	29.1% (41)
- Ph.D.	12.8% (18)
- NA	3.5% (5)
- Other	29.8% (42)

Table 2 shows opinions regarding the development of the Ph.D. program. Participants’ purposes for pursuing a Ph.D. were equally distributed for promotion reasons and research interests. The top 3 factors shaping participants’ decision regarding a Ph.D. program are duration (69.5%), SCFHS recognition (48.9%), and the possibility of being part-time students (45.4%). In terms of transferable skills that should be acquired during a Ph.D., a significant majority (80.1%) emphasized the importance of learning new research technology, followed by gaining publication experience (76.6%). Lastly, the qualities that make a university’s Ph.D. program appealing to participants include good research facilities (86.5%), availability of research funding (74.5%), and a diverse staff of Ph.D. holders (73.8%).

**Table 2:** Opinion Regarding Ph.D. in Endodontic

Variables	% (*N*)
**Purpose of pursuing a Ph.D. degree**	
- Academic job requirement	51.1% (72)
- Interest in research	50.4% (71)
- Pursue promotion in SCFHS reclassification	46.1% (65)
**Considerations before registering for a Ph.D. program**	
- Duration	69.5% (98)
- SCFHS recognition	48.9% (69)
- Being able to be part time	45.4% (64)
- Cost	39.7% (56)
- Accepts non-Saudis	5.7% (8)
**Transferable skills need to be learned during Ph.D.**	
- Learning new research technology	80.1% (113)
- Publication experience	76.6% (108)
- Grants writing	48.9% (69)
- Enhance clinical training	46.8% (66)
**Considerations for choosing a particular university**	
- Good research facilities	86.5% (122)
- Research funding availability	74.5% (105)
- Diversity of Ph.D. staff members	73.8% (104)

Table 3 presents the demographic and professional characteristics of probable prospective Ph.D. candidates in Endodontics. A higher percentage of probable prospective students were in the younger age groups of 20–30 years and 31–40 years (41.4% each) (*P* = 0.001). Gender distribution was balanced with equal representation of males (50.0%) and females (50.0%), with no significant difference found. In terms of the current level of dental education, a significant number had board certification (35.7%) and Endodontic residents/Master students (28.6%) (*P* = 0.006). Regarding SCFHS classification, many probable prospective students were classified as general dentists (37.1%) and consultants (32.9%) (*P* = 0.013). In terms of place of work, the majority were employed at government hospitals (57.1%) and government universities (32.9%), with no significant difference found. Additionally, a considerable proportion of probable prospective students had less than 5 years of expertise (45.7%) (*p* = 0.013), and the highest degree held by many was a Bachelor’s degree (35.7%) (*P* = 0.007).

**Table 3:** Prospective Ph.D. Candidates in Endodontics: Demographic and Professional Characteristics

Variables	Probable prospective student (*N* = 70) % (N)	No (*N* = 71) % (*N*)	*P*-value*
**Age**			0.001
- 20–30	41.4% (29)	14.1% (10)	
- 31–40	41.4% (29)	46.5% (33)	
- 41–50	10.0% (7)	25.4% (18)	
- 51–60	7.1% (5)	14.1% (10)	
**Gender**			0.558
- Male	50.0% (35)	54.9% (39)	
- Female	50.0% (35)	45.1% (32)	
**Your current position**			0.006
- Undergraduate student	5.7% (4)	4.2% (3)	
- General dentist	7.1% (5)	4.2% (3)	
- Endodontic resident/Master student	28.6% 20	5.6% 4	
- Master’s degree	21.4% (15)	25.4% (18)	
- Board certification	35.7% (25)	54.9% (39)	
- Doctorate degree	1.4% (1)	5.6% (4)	
**What is your current SCFHS classification**			0.013
- General dentist	37.1% (26)	14.1% (10)	
- Registrar	10.0% (7)	15.5% (11)	
- Senior registrar	12.9% (9)	8.5% (6)	
- Consultant	32.9% (23)	54.9% (39)	
- NA	7.1% (5)	7.0% (5)	
**Place of work**			0.156
- Government University	32.9% (23)	39.4% (28)	
- Private University	0	5.6% 4	
- Private hospital	10.0% (7)	8.5% (6)	
- Government hospital	57.1% (40)	46.5% (33)	
**Years of expertise**			0.013
- < 5 years	45.7% (32)	25.4% (18)	
- < 10 years	18.6% (13)	14.1% (10)	
- 10–15 years	25.7% (18)	29.6% (21)	
- 16–20 years	2.9% 2	14.1% (10)	
- > 20 years	7.1% (5)	16.9% (12)	
**Highest degree**:			0.007
- Bachelor’s degree	35.7% (25)	14.1% (10)	
- Masters	30.0% (21)	28.2% (20)	
- Ph.D.	5.7% (4)	19.7% (14)	
- NA	1.4% (1)	5.6% (4)	
- Other	27.1% (19)	32.4% (23)	

*Chi-Square Test

**Fig. 1:**
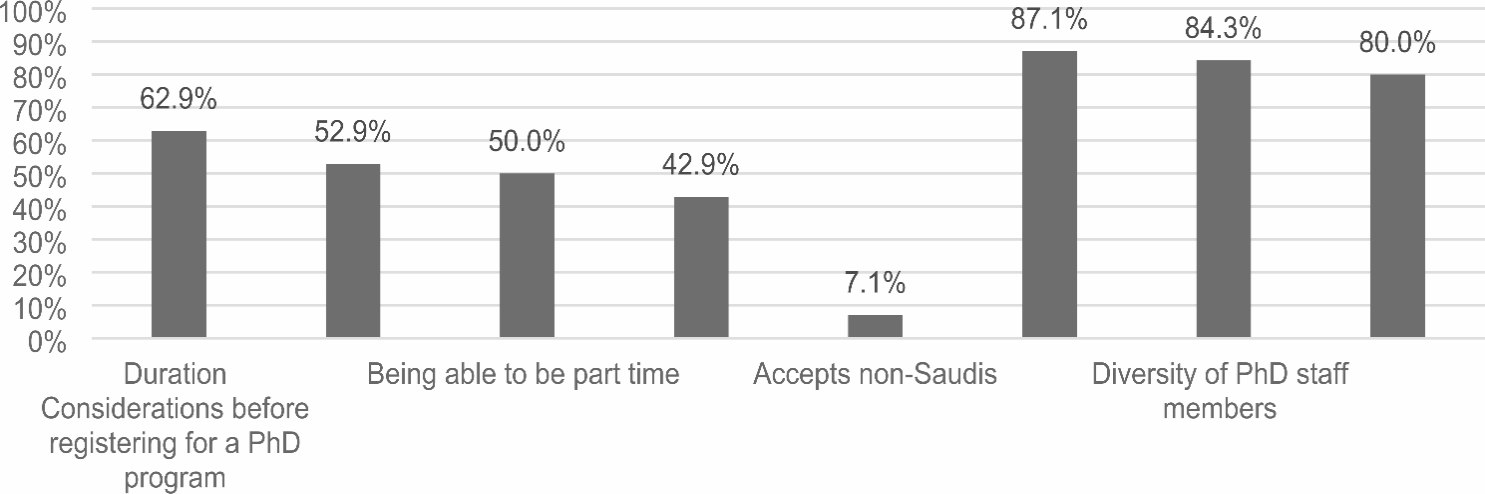
Key considerations for probable prospective Ph.D. students in endodontics

The key considerations for probable prospective Ph.D. students in Endodontics are shown in Fig. 1. When considering registering for a Ph.D. program, 62.9% of students highlighted program duration, 52.9% noted SCFHS recognition, 50.0% valued the ability to be part-time, and 42.9% considered the cost important. Only 7.1% considered the acceptance of non-Saudis. When choosing a particular university, the majority prioritized good research facilities (87.1%), diversity of Ph.D. staff members (84.3%), and research funding availability (80.0%).

**Fig. 2:**
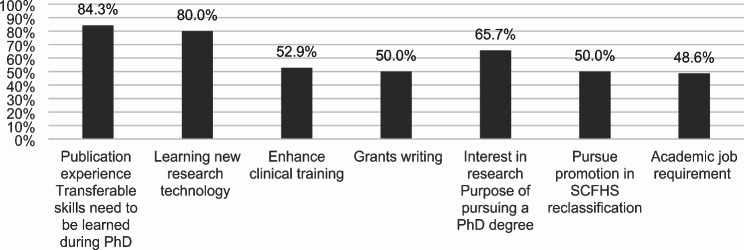
Key transferable skills and purposes for pursuing a Ph.D. among probable prospective students

For transferable skills needed during a Ph.D., 84.3% of probable prospective students emphasized the importance of gaining publication experience, and 80.0% prioritized learning new research technology. Enhancing clinical training was important for 52.9%, and 50.0% valued grant writing. Regarding the purpose of pursuing a Ph.D., 65.7% expressed interest in research, 50.0% aimed to pursue promotion in SCFHS reclassification, and 48.6% cited academic job requirements. These insights are presented in Fig. 2.

Table 4 details opinions on the need for a Ph.D. degree in Endodontics. Candidates who see a need for more Ph.D. degree holders in academic institutions report the highest agreement, with a mean score of 4.67 ± 0.58. This is followed by the general need for a Ph.D. degree program in Endodontics in Saudi Arabia, with a mean score of 4.46 ± 0.83. The need for more Ph.D. holders in the private sector has a mean score of 3.81 ± 1.21, and governmental hospitals show the least agreement with a mean score of 3.74 ± 1.18. All results are statistically significant with p-values < 0.0001.

**Table 4:** Opinions on the Need for a Ph.D. in Endodontics Among Prospective Ph.D. Candidates

Variables	Prospective Ph.D. Candidates in Endodontics	*P*-value*
In Saudi Arabia, there is a definite need for a Ph.D. degree program in Endodontics	4.46 ± 0.83	< 0.0001
Governmental hospitals need more Ph.D. degree holders	3.74 ± 1.18	< 0.0001
Academic institutions need more Ph.D. degree holders	4.67 ± 0.58	< 0.0001
Private sectors need more Ph.D. degree holders	3.81 ± 1.21	< 0.0001

*P-value of Mann-Whitney U test

## Discussion

The current landscape of dental education in Saudi Arabia offers several postgraduate programs in Endodontics, primarily through the Saudi Commission for Health Specialties (SCFHS) and university-affiliated residency programs. However, there remains a significant gap in research-based doctoral programs, specifically a Ph.D. in Endodontics. This gap raises crucial questions about the future direction of Endodontics education in Saudi Arabia and the role of research in advancing the specialty. The development of a Ph.D. program in Endodontics is debatable based on the need for such a program. Moreover, the career choice of dental students is often affected by several factors, depending on their perspective of higher education and their goals in life.

According to the General Authority for the Statistics Demography survey of 2016, the total population of Saudi Arabia is 31,742,308 and it has 410 licensed endodontists, 62% of whom are non-Saudi [31]. This study reflects the demand for highly trained Endodontists in Saudi Arabia.

The Ministry of Education in Saudi Arabia outlines the national qualification framework for all academic programs in the country, including graduate and postgraduate dental programs [32]. This framework was made to meet nationally and internationally qualified dental graduates. Dental graduates are committed to lifelong dental education to meet the requirements of Saudi Licence registration [17]. The development of higher education programs is mandatory to provide dental schools in Saudi Arabia with highly trained Endodontists capable of teaching the specialty and able to design graduate and undergraduate Endodontic programs and courses [33].

The demand for highly trained Endodontic educators is reflected in the difficulty in treating molar teeth in undergraduate students. Studies show that undergraduate students have confidence issues when performing root canal treatment [34, 35]. In Saudi Arabia, dental students reported a high level of stress during their clinical training [36, 37]. The Endodontic procedure, particularly, was one of the most stressful clinical events in dental training [38]. Tanalp et al. suggest that students are less confident in treating complex cases because they tend to refer these cases to postgraduate residents [39]. These findings were also found internationally [39–41]. Highly qualified Endodontic faculty members with a higher degree of education are essential for training graduate and undergraduate dental students and in establishing Endodontic curricula [33] that meet international standards such as Commission on Dental Accreditation (CODA), the European Society of Endodontology (ESE), and the Australian and New Zealand Academy of Endodontists [42–44].

A total of 141 participants responded to this questionnaire regarding the need for a Doctor of Philosophy degree in Endodontics in Saudi Arabia. The majority of responses were particularly from younger age groups (20–30 and 31–40 years) with almost equal gender distribution between males (52.5%) and females (47.5%). This is in line with broader trends seen in the Middle East and globally, where specialized education and research training are becoming essential for career advancement [27]. The responses show that the majority of the respondents were board-certified, classified as consultants by SCFHS, and worked in governmental hospitals. In particular, participants highlighted the importance of Ph.D. holders in academic institutions, suggesting a clear demand for such qualifications to fill teaching and research positions. The demand is not limited to academia, as respondents also recognized the need for more Ph.D.-trained professionals in governmental hospitals and the private sector, albeit to a lesser degree. Two different studies were done at King Saud University to assess the career characteristics and postgraduate education of male and female dentists who graduated from the same university. Among female dentists, 54% completed their postgraduate education, from which 5% obtained their degree in Endodontics [25]. Among male dentists, 77% completed their postgraduate education, from which 7.7% obtained their degree in Endodontics [24]. In a study done by puryer et al., 2016, it was observed that 37.95 dental students in the UK preferred to specialize, with 24.1% choosing restorative dentistry and 20.7% choosing orthodontics. In this study, 79% of the responses were from females [23]. A study by Halawani et al., 2016, on dental specialty career preferences and their influencing factors among final-year dental students in Saudi Arabia showed that the most preferred specialties were restorative and aesthetic dentistry (17.7%), followed by Endodontics (14.1%) and then Prosthodontics (11.7%). The most important factors in choosing a specialty were family influence, preference for private practice, and specific interest in the patient population [45].

In our study, 41% of the age group 20–30 years and 41% of the age group 31–40 years consider themselves as a probable candidate for a Ph.D. program in Endodontics, which reflects the importance of developing such a program. However, it is important to understand the perception of dental students toward the Endodontic specialty. This is a key factor in developing postgraduate programs. Several studies have been done on the career perspective of dental students. A study was done by Alrashdan et al., 2018 on the career perspectives of senior dental students. The data obtained from the results about the choice of career revealed that the majority of the respondents wanted to join specialization courses. These results are observed in a study done by Rashid et al., 2013 [46]. On the other hand, in another study performed in Jordan, it was observed that most of the respondents preferred private practice [26].

In a study by AlMaslamani et al., 2023, at Ajman University, dental undergraduate students were asked about their perception of the Endodontic specialty. Among the respondents, 47.3% expressed their interest in pursuing an Endodontic specialty, with 55.95% due to career interest, 20.6% due to high patient flow and monetary gain, and 20.1% due to county/city need [20]. A study performed in the Middle Eastern region by Halawany et al., 2014 shows that Endodotnics was one of the preferred specialty choices. The study concluded that the career motivation of dental students in Saudi Arabia is associated with socioeconomic aspects, and the future of dentistry is associated with postgraduate education [47]. In a study done by El-Housseiny et al. in 2014 on factors Motivating Career Choices for Dental Students, it was found that grades, family influence, and economic factors with females are more affected by family influences [48].

In Saudi Arabia, motivations for pursuing a Ph.D. are closely tied to career advancement, particularly regarding SCFHS classification and academic job opportunities. Half of the respondents indicated that they seek Ph.D. qualifications to meet academic job requirements, while others expressed interest in research and grant writing as key drivers [49]. This aligns with findings from international studies, where dental professionals increasingly recognize the value of research literacy and publication experience in enhancing their careers. A study in the UAE, for example, found that 28% of dental students were inclined toward pursuing a specialization in Endodontics, and a majority felt that Ph.D. programs opened up opportunities for research and teaching. Most of the respondents (64%) felt that medical universities should open Ph.D. programs as it opens multiple opportunities in teaching and research [19]. A study in the United States of America by Herzog et al., 2018 on Ph.D. program enrollment shows that many Ph.D students can secure the National Institute of Health grants along with dental school funding streams [50]. The study analyzes the enrollment, graduation, and placement outcomes of PhD students from 1994 to 2016, using data from the American Dental Association (ADA) and surveys of 22 oral sciences PhD programs. More females (54.7%) than males (44.5%) were enrolled in PhD programs [50]. In a study by Andriole and Jeffe, 2016, it was shown that 52.5 MD-Ph.D. graduates between (2000–2005) held full-time academic faculty positions by 2013 [51].

Endodontic research has been showing tremendous growth in the dental community in Saudi Arabia. A study by Alrubaig et al., 2023, on Endodontic research performance in Saudi Arabia in the period from 2010 to 2022 shows a growth tendency from 1.29% in 2012 to 7.6% in 2022 from a global perspective. King Saud University was the most prolific institution, and most publications were published in the Journal of Endodontics [52]. In our study, the key consideration that affects a candidate’s choice of joining a Ph.D. program were mainly the availability of good research facilities, staff diversity, and research funding. This further justifies the need for a structured Ph.D. program that can cultivate research skills and provide an academic framework for future endodontists to contribute to scientific advancements.

In Saudi Arabia, the interest in joining postgraduate education programs in dentistry is usually governed by job promotion in the governmental sector. In our study, around 50% of the responses showed that the key factors in pursuing a Ph.D. program are the promotion in SCFHS classification and academic job requirements. Other 50% of the respondents showed interest in learning grant writing.

In a study by Khami et al., 2008, five motive dimensions affected career choices for Iranian dental students and they were altruism and intellectual challenges, characteristics of the profession, social status and security, other person’s recommendation, and failure to be admitted to other study programs [53]. Similarly, a study was done in Saudi Arabia on the associated influences and motivating factors in choosing a dental specialization. These factors were found to be job security and financial stability, encouragement from others, the nature of dentistry as a profession, family encouragement, interactions with dentists, and work-life balance flexibility [54]. This study identifies several key factors that influence prospective students’ decision to pursue a Ph.D. in Endodontics. The program’s duration, recognition by SCFHS, and the ability to study part-time were critical considerations for participants. These practical concerns suggest that any Ph.D. program in Endodontics must offer flexible options to accommodate professionals already engaged in clinical practice. Furthermore, the availability of good research facilities, a diverse staff of Ph.D. holders, and research funding are essential to attracting high-caliber candidates.

Steps should be taken to motivate dentists to pursue Ph.D. degrees. The emphasis on research infrastructure is particularly important in the context of the rapid technological advancements in Endodontics, such as new diagnostic tools, treatment techniques, and materials. Ph.D. programs are expected to equip candidates with the skills to critically evaluate these advancements and contribute to their development. As noted by previous studies, Endodontics is increasingly influenced by evidence-based practices, and Ph.D. holders will play a pivotal role in driving innovation and maintaining the highest standards of care. Candidates who are interested in pursuing a Ph.D. degree in Endodontics should focus on specific skills such as critiquing articles, writing scientific papers, and curricular development.

## Conclusion

This study demonstrates a significant need for a Ph.D. program in Endodontics within Saudi Arabia. The high value placed on factors such as program duration, SCFHS recognition, and part-time options underscores the need for a program design that accommodates the needs of practicing professionals. Furthermore, the emphasis on research infrastructure, including readily available research funding, advanced facilities, and a diverse faculty, reflects the importance of establishing a research-focused program to address the rapidly evolving landscape of.

Endodontics. Future researches are needed such as longitudinal studies on the career trajectories of Ph.D. holders in endodontics.

## Limitations of the study

The representative sample size of the participants of the population of dental professionals in Saudi Arabia is small. Response bias of participants such as overestimating or underestimating their interest in a ph.D. due to social desirability or perceived expectations. The study focused on a limited number of institutions and this will affect the generalization of the results. Further studies should be done to compare the skills gained different doctoral programs in both the dental and the medical fields. Moreover, further studies could compare the research productivity in schools with postgraduate programs in comparison to schools that do not have such programs.

## Electronic Supplementary Material

Below is the link to the electronic supplementary material.

Supplementary Material 1

## Data Availability

The datasets used and/or analyzed during the current study are available from the corresponding author upon reasonable request. Data cannot be deposited in a public repository since this research is not yet published. For that reason, and data confidentiality data will be granted upon reasonable request.

## Abbreviations

Ph.D.: Doctor of Philosophy
KAUFD: Faculty of Dentistry King Abdulaziz University
UAE: United Arab Emirates
CBCT: Cone Beam Computed Tomography
KSU: King Saud University
SBE: Saudi Board in Endodontics
