# Supplementary material for: Assessing the need for a doctor of philosophy (Ph.D.) degree in Endodontics: perspective and implication for advancing dental education and research in Saudi Arabia
Source: BMC Med Educ. 2024 Dec 18;24:1429. doi: 10.1186/s12909-024-06485-w (PMC11654090; doi:10.1186/s12909-024-06485-w)
Supplement: Supplementary file 1 — Supplementary Material 1 [file 12909_2024_6485_MOESM1_ESM.docx]

**PhD in Endodontics survey**

This questionnaire aims to evaluate the need for PhD in Endodontics programs in Saudi Arabia. The survey is directed toward both prospective candidates and decision-makers

Please note the following:

The PhD program in Endodontics will be designed to have both clinical and research

components. The PhD program in Endodontics will require candidates to have a

MSc in Endodontics degree.

What is your age group?

- 20-30
- 31-40
- 41-50
- 51-60

What is your gender?

- Male
- Female

What is your current level of dental education?

- Undergraduate student
- Bachelor’s degree
- Endodontic resident/Master student
- Master’s Degree
- Board Certification
- Doctorate Degree

What is your current SCFHS classification?

- General Dentist
- Registrar
- Senior registrar
- Consultant
- NA

What is your current place of work?

- Government University
- Private University
- Private hospital
- Government hospital

Do you see yourself as a prospective student for PhD degree in Endodontics?

- Yes
- No
- NA

How many years of professional experience do you have?

- <5 years
- <10 years
- 10-15 years
- 16-20 years
- >20 years

What is your highest academic degree?

- Bachelor's degree
- Masters
- PhD
- NA
- Other:

Will you be interested in acquiring Ph.D. degree in endodontics?

- Yes
- No
- NA

What is your purpose of pursuing a PhD degree parallel with your endodontic specialty?

- Academic facility requirement
- Interested in research
- Pursue a higher degree to reclassify for SCFHS

What characteristics/factors would you consider before applying for a Ph.D. program?

- Duration
- Cost
- SCFHS recognition
- Being able to be a part time student
- If it accepts non-Saudis

Please rate the following on a scale from strongly agree to strongly disagree:

|  | Strongly agree | Agree | Neutral | Disagree | Strongly disagree |
| --- | --- | --- | --- | --- | --- |
| In Saudi Arabia, there is a definite need for a PhD degree program in Endodontics. |  |  |  |  |  |
| Governmental hospitals need more PhD degree holders |  |  |  |  |  |
| Academic institutions need more PhD degree holders |  |  |  |  |  |
| Private sectors need more PhD degree holders |  |  |  |  |  |

What transferable skills should one learn during the PhD? *

- Publication experience.
- Grants writing.
- Learning new search technology.
- Enhance clinical training

What makes a particular school in the University good for your Ph.D. studies? *

- Good research facilities
- Diversity of staff members (Ph.D. holders)
- Research funding availability
